# Expression of TFRC helps to improve the antineoplastic effect of Ara-C on AML cells through a targeted delivery carrier

**DOI:** 10.1186/s12951-023-01881-8

**Published:** 2023-04-11

**Authors:** Xinzhou Wu, Zhouguang Jiao, Junying Zhang, Feng Li, Yuhua Li

**Affiliations:** 1 Department of Hematology, Zhujiang Hospital, Southern Medical University, Guangzhou, People’s Republic of China; 2 State Key Laboratory of Biochemical Engineering, Institute of Process Engineering, Chinese Academy of Sciences, Beijing, 100190 People’s Republic of China

**Keywords:** Acute myeloid leukemia, Transferrin receptor, Ferritin, Targeted delivery, Ara-C

## Abstract

**Background:**

Currently, high doses of cytarabine arabinoside (Ara-C)-based combined chemotherapy are commonly used in acute myeloid leukemia (AML) therapy, but severe adverse effects and poor suppression effects in leukemia cells limit the clinical therapeutic efficiency of Ara-C-based chemotherapy due to a lack of targeting selectivity. To improve the therapeutic effect of Ara-C in AML, here, since we confirmed that transferrin receptor 1 (TFRC) expression in AML cells was constant, we generated Ara-C@HFn by encapsulating free Ara-C into self-assembled heavy ferritin chain (HFn, the ligand of TFRC) nanocages.

**Results:**

The analysis of clinically relevant data suggested that the high expression levels of TFRC from AML cells would not decrease significantly after treatment with Ara-C. Ara-C@HFn can be efficiently internalized by leukemia cells, showing stronger cytotoxic effects in vitro and reducing the burden of leukemia in AML mice more effectively in vivo than free Ara-C. Ara-C@HFn treatment showed no acute toxicity in visceral organs of mice. Moreover, the analysis of clinically relevant data also suggested that there are several drugs (such as tamibarotene and ABT199) that would not cause significant expression down-regulation of TFRC in AML cells (after treatment).

**Conclusion:**

The above results suggested that TFRC can be used as a constant and effective target for drug targeting delivery of AML cells. Thus Ara-C@HFn treatment can become a safe and efficient strategy for AML therapy by specifically delivering Ara-C to AML cells. Besides, the HFn nanocages are promising for improving antineoplastic effect of other AML-related therapy drugs that do not cause downregulated expression of TFRC in AML cells.

**Graphical Abstract:**

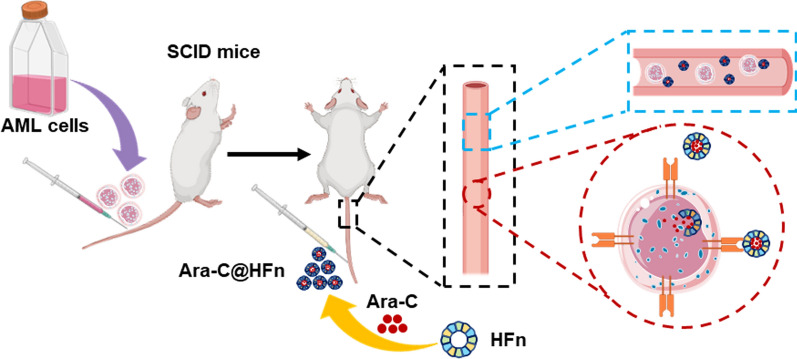

**Supplementary Information:**

The online version contains supplementary material available at 10.1186/s12951-023-01881-8.

## Background

As a highly heterogeneous hematological malignancy, acute myeloid leukemia (AML) is characterized by immature myeloid cell proliferation and bone marrow failure [1]. AML accounts for 15–20% of acute leukemia cases in children and approximately 80% in adults [2], and the 7 + 3 regimen (i.e., continuous infusion of cytarabine arabinoside for 7 days with three once-daily injections of an anthracycline) for AML induction therapy has been used for more than 40 years [3]. Despite the fact that patients' lives can often be prolonged with proper treatment, the outcomes of patients with AML are usually described as “dire” or “poor” [4]. In fact, as the most effective drug against AML, the free pyrimidine analog cytarabine arabinoside (Ara-C) used in the clinic can be widely distributed in the body even in off-target areas; thus, a high dose of Ara-C has to be used to achieve an ideal curative effect in AML patients [5]. The high dose of Ara-C can kill normal hematopoietic cells, which puts patients at risk of suffering some complications, such as infections, inflammation, cardiotoxicity and even death [6, 7]. Therefore, it is imperative to develop new drug delivery strategies for improving the efficiency of Ara-C uptake by leukemic cells to reduce the severe adverse effects induced by Ara-C and to achieve better curative effects in AML.

To improve the uptake efficiency of Ara-C, as well as reduce drug-related complications, targeted delivery is a particularly attractive method that can improve the local effective anticancer drug concentration at target cells [8]. The prerequisite of a targeted delivery system is identifying a specific and stable target on the surface of leukemia cells. Iron uptake by cells occurs primarily through transferrin receptors (TFRs) [9]. As the best-characterized receptor for human transferrin, TFRC (TfR1) is a type II transmembrane glycoprotein and comprises two homologous subunits (90 kDa) that are joined by disulfide bonds, with a small cytoplasmic domain and a large extracellular domain [9, 10]. In general, TFRC is expressed at low levels on most normal cells in humans [11–13]. Increased expression is usually observed on cells with a high rate of proliferation and with a high need for iron, such as cancer cells [14, 15]. The density of TFRC at the cell surface can be greatly increased when cells become malignant [16], and previous research has proven that the TFRC could be a therapeutic target for AML since TFRC has higher expression levels on AML cells than on normal body cells [17].

As the TFRC ligand [18], ferritin is a naturally spherical iron storage protein comprising 24 self-assembled subunits of two types, the heavy (H) and light (L) chains, with a ratio that varies drastically [15, 19, 20]. Ferritin protein self-assembles spontaneously in the physiological environment into a hollow nanocage with an outer diameter of 12 nm and an interior cavity with a diameter of 8 nm [21], thus allowing the rapid expression of functional ferritin in prokaryotic systems [22]. The inner cavity could provide potential space to encapsulate small molecule drugs as well as offer protection from the degradation of drug molecules [15, 17, 19, 20]. In vivo, the ferritin H chain can be recognized by TFRC and taken up by cells [18]. Unlike conventional synthetic or modified materials, ferritin is a natural protein in the body and therefore is biocompatible and nonimmunogenic [19, 20]. In addition, despite their rigidity under physiological conditions, the ferritin nanocages can disassemble into subunits in an acidic environment (e.g., pH = 2) and be reconstituted into a nanocage structure when the pH returns to neutral (*e.g.*, pH = 7.4) [23–25]. In general, the above characteristics of ferritin, especially ferritin nanocages assembled with only the H ferritin chain (HFn), endow it with considerable prospects for application in AML targeted therapy.

**Scheme 1:**
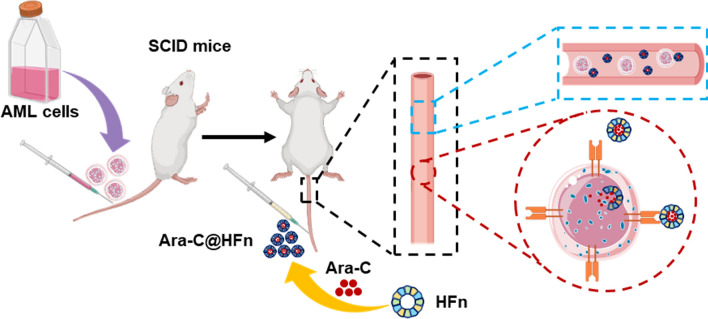
Schematic illustration of AML leukemia development suppression through Ara-C@HFn

Although it is known that TFRC has a high expression level in cancerous cells in AML patients, whether the expression of TFRC in AML cells decreases after treatment with Ara-C has not been reported; the downregulated expression of TFRC will interfere the long-term effectiveness of targeted therapy based on TFRC. In this context, this study first analyzed some sequencing data of related patients’ sample from the Gene Expression Omnibus (GEO) database and concluded that the expression of TFRC in AML cells would not significantly decrease after Ara-C treatment. These findings further encouraged us to develop an HFn nanocarrier loaded with Ara-C (Ara-C@HFn) in the ferritin cavity. The nanocarrier can be specifically recognized by AML cells and can rapidly release Ara-C into leukemia cells following intravenous injection, which has the potential to enhance the therapeutic effect and reduce the side effects to normal body cells induced by Ara-C (Scheme 1).

## Materials and methods

### Cell lines

HL-60 (1101HUM-PUMC000037) and K562 (1101HUM-PUMC000039) cells were sourced from the National Infrastructure of Cell Line Resource. No mycoplasma contamination was detected in the above cell lines. The cells were maintained in RPMI-1640 media (Biological Industries, Kibbutz Beit-Haemek, Israel) supplemented with 10% fetal bovine serum (FBS, Gibco), 100 U/mL penicillin, and 10 mg/mL streptomycin (Solarbio, Beijing, China) at 37 °C with 5% CO_2_. HL-60 cells transduced with luciferase were used in some experiments as indicated.

### Animals

B-NDG (NOD-*Prkdc*^*scid*^*IL2rg*^*tm1*^/Bcgen) severe combined immune deficiency (SCID) mice that were female and 5–6 weeks old were purchased from Beijing Biocytogen Co., Ltd. Specific pathogen‑free (SPF) female BALB/c mice (6‑8 weeks old) were obtained from Vital River Laboratory (Beijing, China). Mice were maintained in SPF conditions with a 12 h/12 h light/dark cycle, and the mice received food and water ad libitum. This study was performed in strict accordance with the Regulations for the Care and Use of Laboratory Animals and Guideline for Ethical Review of Animals (China, GB/T 35892-2018). All animal experiments in this study were reviewed and approved by the Animal Ethics Committee of the Institute of Process Engineering (IPEAECA2021081).

### Preparation and characterization of Ara-C@HFn

Human HFn comprising all heavy-chain subunits of ferritin was produced and purified in *E. coli* as previously described [17]. The loading of Ara-C into the cavity of HFn was carried out according to published procedures with minor modifications [26, 27]. Briefly, HFn (8 mg) was dispersed in 20 mL of PBS buffer (pH 7.4) and disassembled into subunits by adjusting the pH to 2.0 with HCl. Then, 5 mL of 30 mg/mL Ara-C (Aladdin) was added to the HFn solution. After incubation for 10 min, the pH was returned to 7.4. The mixture was gently stirred at room temperature for 30 min and purified on an ultrafiltration membrane column (molecular weight cutoff 10 kD, Millipore) for at least 5 times to remove free Ara-C. And the Ara-C can not be detected in the last filtrate. The concentration of HFn was determined using a bicinchoninic acid (BCA) assay kit (Thermo Fisher Scientific).

The concentration of Ara-C was analyzed with reversed-phase high performance liquid chromatography (RP-HPLC, Shimadzu SIL-20A, Kyoto, Japan) using a C18 column (4.6 mm × 250 mm, 5 μm particle size, DIKMA, Beijing, China) with a linear gradient of acetonitrile containing 0.1% TFA from 0 to 100% in 10 min at a flow rate of 1.0 mL/min. Initially, the peak area of the Ara-C standard with various concentrations was detected by HPLC, and the standard curve was fitted accordingly. The Ara-C concentration in Ara-C@HFn was determined by HPLC, and the levels of Ara-C encapsulated by HFn were measured according to the concentration of HFn (approximately 56 Ara-C molecules were encapsulated by one HFn nanocage).

The blank HFn nanocage, disassembled HFn nanocage (pH = 2.0), and Ara-C@HFn were prepared for transmission electron microscopy (TEM) by directly dropping these aqueous solutions on 400 mesh ultrathin carbon film (Beijing Zhongjingkeyi). These samples were then negatively stained with uranyl acetate and observed by TEM (operated at 120 kV, JEM-1400 Flash, n = 3).

The size distributions of HFn and Ara-C@HFn were determined by dynamic light scattering (DLS, Malvern) in a Nano series. Samples were tested in triplicate, and the average peak size (nm) was recorded (n = 3).

The molecular weight of the protein subunit from Ara-C@HFn, HFn using the same amount of protein as that in Ara-C@HFn, and Ara-C using the same amount of Ara-C as that in Ara-C@HFn was determined by SDS–polyacrylamide gel electrophoresis (SDS‒PAGE) using a standard operation procedure (n = 3).

### Cell internalization analysis

Confocal laser scanning microscopy (Nikon) was used to observe the internalization of Ara-C@HFn. Before observation, Cy5-labeled Ara-C@HFn was prepared. Briefly, Cy5NHS ESTER (Meilunstar) was dissolved in water and incubated with Ara-C@HFn at a molar ratio of more than 20:1. The mixed solution was stirred at 4 °C overnight, and the free dye was removed by ultrafiltration. Then, HL-60 or K562 cells were incubated with Cy5-labeled Ara-C@HFn for 24 h at 37 ℃ (in RPMI-1640 media) and fixed in 4% cold formaldehyde for approximately 15 min. Subsequently, the cell membrane was stained with rhodamine phalloidin (Invitrogen) for 30 min, and the nucleus was stained with Hoechst 33342 (Solarbio) for 20 min. Finally, the prepared samples were observed with a confocal laser scanning microscope (Nikon) by NIS Elements AR 5.20 (n = 3). Note that for lysosomal staining, it is not necessary to fix the cells before staining. After incubating the lyso-Tracker Green (Beyotime) and Hoechst 33342 (Solarbio) with cells for 20 min at 37 ℃, then the prepared samples could be observed with a confocal laser scanning microscope.

### Cell viability analysis

The cell viability assay was similar to that used in our previously published paper [28]. Briefly, HL-60 and K562 cells that were seeded into 96-well cell culture plates at a density of 1 × 10^5^ cells/well were treated with PBS buffer, various concentrations of free Ara-C or various concentrations of Ara-C@HFn with the equivalent amount of Ara-C for 48 h. After incubating for 2 h, the absorbance value at 450 nm in each well was measured by the Infinite M200 microplate spectrophotometer (TECAN, Switzerland), and cell viability was assessed by using the CCK-8 assay (Cell Counting Kit, Biosharp, n = 4).

### Antibody blocking assay

An antibody blocking assay was performed according to the published procedures [17, 19] to confirm that TFRC is the binding receptor of Ara-C@HFn to HL-60 cells. Briefly, the HL-60 cells were incubated without or with anti-TFRC antibody (Invitrogen) for 2 h at a dose of tenfold molar excess than the counts of Ara-C@HFn. The internalization and cell viability analysis were performed based on the methods mentioned in the cell internalization analysis and the cell viability analysis sections then.

### In vitro Ara-C release studies

To assay for the release of Ara-C, Ara-C@HFn samples (450 μM Ara-C equivalents, 500 μL/tube) were placed in Eppendorf centrifuge tube and incubated in either PBS buffer (pH 7.4) or hydrochloric acid buffer (pH 5.0) at 37 °C in the dark. After removing the Ara-C@HFn by using ultrafiltration membrane columns (molecular weight cutoff 10 kD, Millipore), the released free Ara-C from Ara-C@HFn at different incubation times was determined by HPLC with a C18 column.

### Cell cycle analysis

HL-60 cells were cultured in 12-well plates at a density of 1 × 10^6^ cells/well and incubated with 0.6 μM Ara-C or Ara-C@HFn with the equivalent amount of Ara-C for 48 h. The cell cycle was assessed using a DNA Content Quantitation Assay (Solarbio) through the flow cytometry system (Beckman, n = 3).

### Evaluation of the targeting capacity of Ara-C@HFn in vivo

The AML-bearing mouse model was established as described in a previously published paper [17]. Briefly, SCID mice were injected with cyclophosphamide (100 mg/kg) intraperitoneally and inoculated with 5 × 10^6^ AML cells intravenously the following day. At 15 days post HL-60 cell injection, the mice were injected with Cy5-labeled Ara-C@HFn, and 2 h later, the peripheral blood, spleen and hindlimbs of the AML-bearing mice were collected to evaluate targeting capacity. Then, the spleen and hindlimbs were used to prepare single-cell suspensions, red blood cells (RBCs) were removed from the peripheral blood and single-cell suspensions by using RBC lysis buffer (Solarbio). Subsequently, these samples were incubated with EV450-conjugated anti-human CD45 (anti-hCD45) antibody (eBioscience) for 30 min, and the proportion of hCD45^+^ cells (the marker of HL-60 cells) in Cy5^+^ (the label of Ara-C@HFn) cells was analyzed by flow cytometry (n = 3).

### Suppression of leukemia cells in vivo

The AML mouse models were established with Luc-HL-60 cells (HL-60 cells that stably expressed luciferase) as described above. All mice were randomly divided into three groups (n = 4): control (Ctrl, mice treated with PBS), free Ara-C and Ara-C@HFn. Four days later, the mice received six-round treatments of PBS, Ara-C (8 mg/kg) or Ara-C@HFn (with the same equivalent dose of Ara-C) (once every 2 days). Then, leukemia progression was monitored by an In vivo Imaging System (IVIS) optical imaging system (PerkinElmer) once every 5 days. AML-bearing mice were injected with D-luciferin potassium (150 mg/kg, Meilunstar) intraperitoneally, and then the bioluminescence signals from AML-bearing mice were determined by Living Image 4.5.2. All mice were euthanized at 20 days after the HL-60 cell transplantation, following which the peripheral blood, backbone, hindlimbs were isolated. The leukemia burden in interested tissues was evaluated by measuring the bioluminescence levels. Subsequently, a small amount of peripheral blood was used for white blood cell (WBC) counting. In addition, most of the peripheral blood and the spine were used to prepare single-cell suspensions, which were stained with EV450-conjugated anti-human CD45 antibody and/or APC-conjugated anti-human TFRC antibody (Biolegend) for subsequent flow cytometry analysis.

### Toxicity assessment

The Balb/C mice were treated with PBS, free Ara-C or Ara-C@HFn (the twice equivalent dose of Ara-C as therapeutic dose). Forty-eight hours later, peripheral blood and the major organs, such as the heart, liver, spleen, lung, and kidney, were collected for acute toxicity assessment. Peripheral blood was used for routine blood tests, and the major organs were used for histopathological analysis (n = 3).

### Histopathology

The histopathological analyses were performed according to standard operating procedures as described in a previously published paper [29]. Briefly, the heart, lung, spleen, liver, and kidney were dehydrated and embedded using paraffin, sliced into approximately 5 μm sections and placed on glass slides. Hematoxylin & Eosin (H&E) staining was then performed on sections from the heart, lung, spleen, liver, and kidney. After staining, the sections from H&E staining were analyzed and evaluated (n = 3).

### Data analysis based on the GEO database

This study further analyzed AML-related transcriptome sequencing data uploaded to the GEO database (http://www.ncbi.nlm.nih.gov/projects/geo/) over the past two years (data uploaded after July 20, 2020). To facilitate further analysis, the inclusion criteria of the data analyzed in this study mainly included the following: the library preparation strategy was for RNA-seq (including single-cell RNA sequencing data); the sequencing data were directly obtained from human or transgenic mice that were treated with transplanted human volunteer cells; and if the transgenic mice were treated with drugs, the drugs were used in the clinic or in clinical trials.

### Statistical analysis

In this study, the statistical analysis for the single-cell RNA sequencing (scRNA-seq) data was performed by R software (v4.1.0), and the statistical analysis of other data was performed by SAS software (v9.3, SAS Institute, Inc.). Among them, for statistical analysis of two groups of data, when the data conformed to a normal distribution and exhibited homogeneity of variance, the t test was used; otherwise, the Wilcoxon rank sum test was used. For statistical analysis of three or more groups of data, when the data conformed to a normal distribution and exhibited homogeneity of variance, one-way ANOVA was used; otherwise, the Kruskal‑Wallis test was performed, and the Student‑Newman‑Keuls test was used for multiple comparisons of data. In addition, these statistical data were plotted using GraphPad Prism (v8.4.3) except for scRNA-seq data, principal component analysis (PCA) and correlation analysis, which were plotted using R software. Note that each experiment in this study has no less than 2 technical replicates.

## Results

### Preparation and characterization of Ara-C@HFn

Previously, based on the expression of TFRC in a large number of AML patient cells assessed by flow cytometry, we concluded that TFRC has a high expression level on AML cells [17]. However, whether the RNA sequencing data are consistent with the conclusions reached from flow cytometry analysis is still uncertain. Whether or not the data in the GEO database are consistent with our previous results based on flow cytometry analysis needs further exploration. To this end, this study first screened RNA sequencing data containing healthy donors and AML patients obtained from the data uploaded in the GEO database in the past two years and analyzed the expression of TFRC in the RNA sequencing data. The results suggested that in the RNA sequencing data obtained from bone marrow endothelial progenitor cells (GSE197907, Additional file 1: Fig. S1a) or bone marrow immune cells (GSE154109, Additional file 1: Fig. S1b) isolated from AML patients or healthy donors, the average TFRC expression in AML patient cells was significantly higher than that in healthy donor cells. This result is consistent with our previous conclusions based on flow cytometry analysis.

Considering the important role of Ara-C in AML treatment and the important advantages of HFn in targeted drug delivery (such as good biosafety and ease of expression and purification), the above analysis results encouraged us to further analyze whether TFRC expression was downregulated in AML cells post Ara-C treatment based on the GEO database. If Ara-C leads to significant downregulation of the expression of the HFn receptor TFRC, HFn-based targeted drug delivery will be difficult to achieve, and the effect of HFn-based targeted drug delivery will be greatly reduced. Although the available effective data are only a little, it is exciting that our analysis results suggested that the expression level of TFRC in AML cells from AML patients was upregulated after Ara-C treatment in vitro (GSE145061, Additional file 1: Fig. S1c) and that the expression of TFRC was transiently downregulated in transgenic mice transplanted with AML patient cells (GSE146592, Additional file 1: Fig. S1d).

**Fig. 1:**
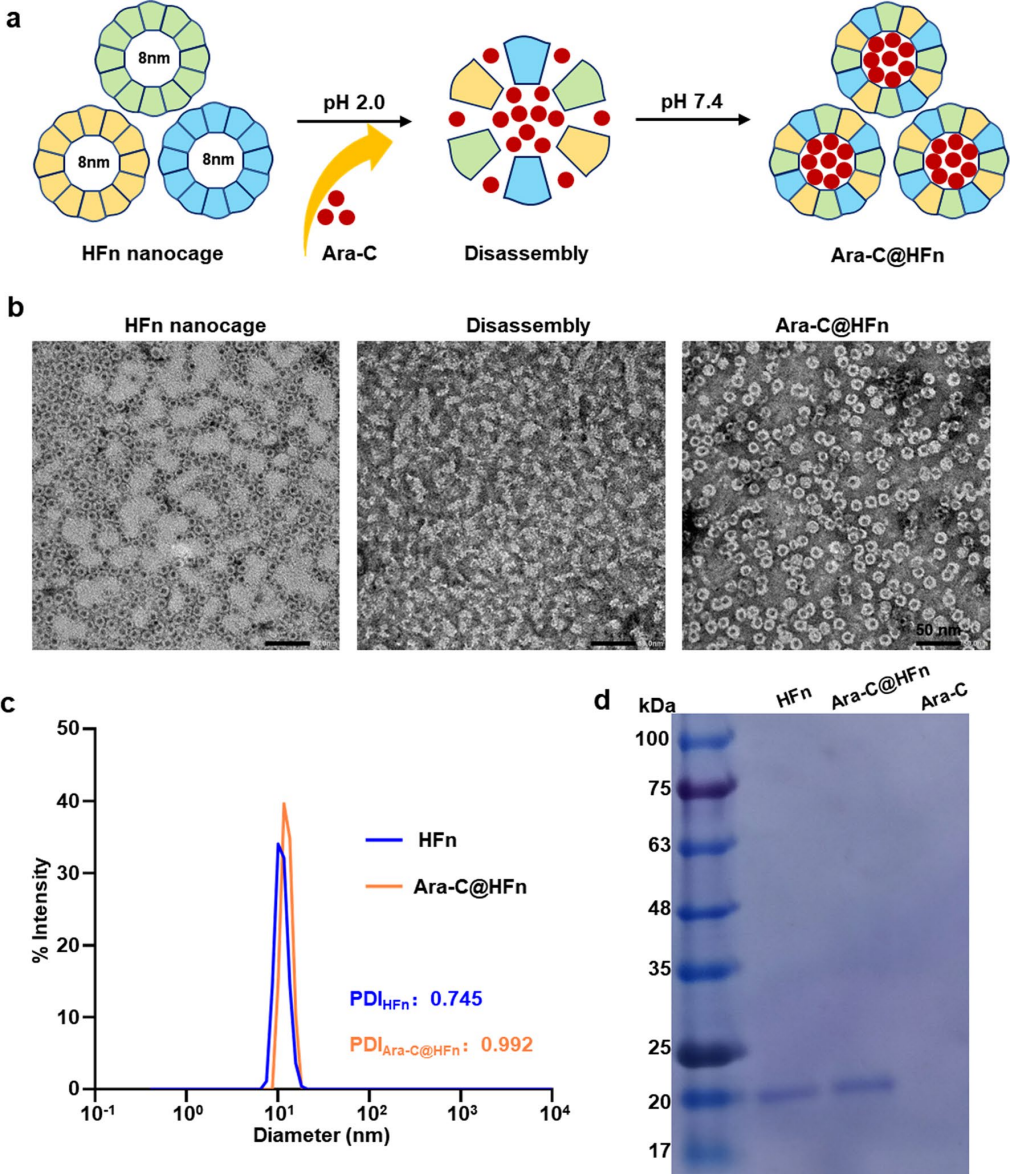
Preparation and characterization of Ara-C@HFn. **a** Schematic depiction of the preparation process used to generate Ara-C@HFn. **b** Representative high-resolution transmission electron microscopy (TEM) images of blank HFn nanocages (left), disassembled HFn nanocages (middle) and Ara-C@HFn. Bars size: 50 nm. **c** The intensity distribution of HFn and Ara-C@HFn. **d** SDS‒PAGE analysis of HFn, Ara-C@HFn and free Ara-C. *PDI* Polydiseperse Index

The above analysis results further motivated us to use HFn to load Ara-C to improve the therapeutic effect of Ara-C in AML. Initially, human HFn was expressed in *Escherichia coli* and purified as previously described [17]. Ara-C@HFn was prepared by encapsulating Ara-C into the cavities of HFn nanocages using the previously reported disassembly/reassembly method [26]. Briefly, HFn was disassembled and incubated with Ara-C in strongly acidic PBS buffer, and then HFn was reassembled in neutral PBS buffer (Fig. 1a). After removing the free Ara-C by ultrafiltration, the levels of encapsulated Ara-C were measured by HPLC, which showed that approximately 56 Ara-C molecules were loaded into each HFn nanocage. The encapsulation efficiency and drug-loading rate of Ara-C@HFn were about 0.15% and 2.72%, respectively. HFn were monodispersed nanocages in neutral solution (pH = 7.4) with a spherical morphology that dissociated into irregular individual subunits in strong acidic solution (pH = 2), and the purified Ara-C@HFn were still monodispersed nanocages with a spherical morphology in neutral solution, as observed by TEM (Fig. 1b). DLS analysis showed that Ara-C@HFn had a narrow size distribution (the Polydiseperse Index is about 0.992), and there was no significant difference observed in peak size between HFn (11.7 nm) and Ara-C@HFn (12.5 nm) (Fig. 1c). In addition, the SDS‒PAGE results suggested that the subunit of HFn from Ara-C@HFn was not degraded due to the rapid change in pH (disassembly/reassembly) in this study (Fig. 1d). The above results together illustrate that the preparation of Ara-C@HFn in this study was successful and demonstrates promise in future applications.

### Internalization and cytotoxicity of Ara-C@HFn

**Fig. 2:**
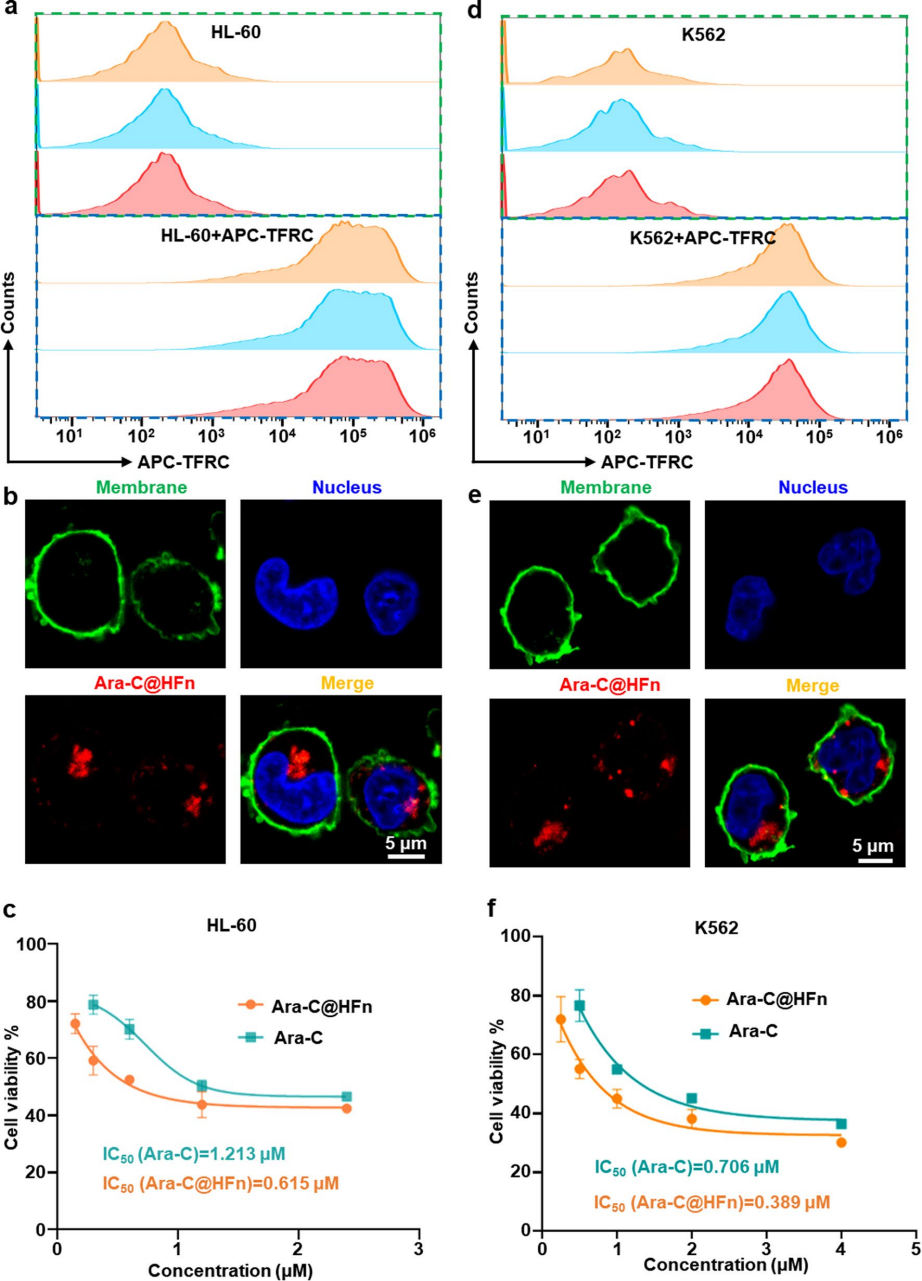
Internalization and cytotoxicity assessments of Ara-C@HFn in leukemia cells. **a**, **d** Flow cytometry analysis of TFRC expression in HL-60 **a** and K562 **d** cells (n = 3). **b**, **e** Representative confocal laser scanning microscope images of Ara-C@HFn (Cy5 labeled) endocytosed by HL-60 **b** and K562 **e** cells (green, cell membrane; blue, nucleus; red, Cy5–Ara-C@HFn; scale bar, 5 μm). **c**, **f** Relative viability analysis of HL-60 **c** and K562 **f** cells after treatment with different concentrations of Ara-C@HFn or free Ara-C for 48 h (n = 4)

Since Ara-C@HFn was successfully prepared, we next evaluated the internalization and cytotoxicity of Ara-C@HFn using the AML cell line, HL-60. To observe the internalization of Ara-C@HFn, we initially determined the expression of TFRC, which is the receptor for HFn and can mediate internalization of Ara-C@HFn, on the surface of HL-60 cells. As expected, flow cytometry analysis showed that AML cells had high TFRC expression levels (Fig. 2a). Then, by incubating Cy5-labeled Ara-C@HFn (Cy5 covalently bound to HFn nanocages) with AML cells, we observed that a large number of HFn molecules were endocytosed into cells through confocal laser scanning microscopy (CLSM) (Fig. 2b), which suggested that HFn nanocages can help to increase the uptake efficiency of Ara-C by leukemia cells.

The above internalization studies of Ara-C@HFn further encouraged us to explore the cytotoxicity of Ara-C@HFn; the aim of this experiment was to demonstrate that, after encapsulation, the pharmacological activity of Ara-C could be preserved. The results of the CCK8 cell viability assay suggested that the IC_50_ (half maximum inhibitory concentration) of Ara-C@HFn was approximately 50% lower than that of free Ara-C (Fig. 2c). This result indicates the potential therapeutic applications of Ara-C@HFn, and as expected, HFn nanocages increase the uptake efficiency of Ara-C, resulting in stronger cytotoxicity.

The above results motivated us to expand our study to leukemia cell lines other than AML cells, such as the chronic myelogenous leukemia (CML) cell line, K562. Flow cytometry indicated that TFRC exhibited a high expression level on the surface of CML cells (Fig. 2d). CLSM observations showed that CML cells could also endocytose HFn (Fig. 2e), and the cell viability results showed that the IC_50_ of Ara-C@HFn was approximately 45% lower than that of free Ara-C (Fig. 2f). These results suggest that the phenomena of HFn nanocage-mediated internalization of Ara-C and enhanced cytotoxicity observed in AML cells did not occur by chance.

In order to confirm that TFRC is the binding receptor of Ara-C@HFn to HL-60 cells, we determined the changes of internalization efficiency and cytotoxicity of Ara-C@HFn through antibody blocking assay by using anti-TFRC antibody. The results suggested that after blocking the TFRC, there were less Ara-C internalized by HL-60 cells, while there was a large amount of Ara-C internalized into lysosomes by the normal HL-60 cells without blocking TFRC (Additional file 1: Fig. S2a, b). In addition, after blocking the TFRC on the surface of HL-60, the cytotoxicity of Ara-C@HFn to HL-60 cells also decreased obviously (Additional file 1: Fig. S2c). The above results suggested that Ara-C can be internalized into the lysosome, which encouraged us to further explore the potentially intracellular release behavior of Ara-C from Ara-C@HFn. We used HPLC to continuously monitor the release behavior of Ara-C from Ara-C@HFn at pH 5.0 and pH 7.4 (used to simulate lysosomal environment and normal physiological environment respectively) at 37 ℃. The results showed that Ara-C could release close to 90% from Ara-C@HFn when incubated at pH 5.0 solution at 80 h, while Ara-C did not release obviously from Ara-C@HFn at pH 7.4 over 80 h (Additional file 1: Fig. S2d).

### Targeting ability of Ara-C@HFn in vivo

Although the results of cell cycle analysis suggested that Ara-C@HFn and free Ara-C exhibited similar S and G_2_/M arrest characteristics in AML cells (Additional file 1: Fig. S2e, f), considering that the Ara-C concentration was excessive in the in vitro experiment and all cells exhibited contact with the Ara-C drug directly, the difference in the effects of targeted drugs (Ara-C@HFn) and nontargeted drugs (Ara-C) could be reduced. Therefore, the above results regarding the internalization and cytotoxicity of Ara-C@HFn prompted us to further investigate the targeting ability of Ara-C@HFn in vivo. We established the HL-60 leukemia mouse model based on SCID mice, and the peripheral blood, spleen and hindlimbs were collected to evaluate targeting capacity by flow cytometry after treatment with Cy5-labeled Ara-C@HFn. The results showed that more than 80% of the cells that internalized Ara-C@HFn (Cy5^+^ cells) were leukemia cells (hCD45^+^ cells) (Additional file 1: Fig. S3a, b), which proved the excellent targeting ability of Ara-C@HFn.

### Suppression of AML leukemia development in vivo

**Fig. 3:**
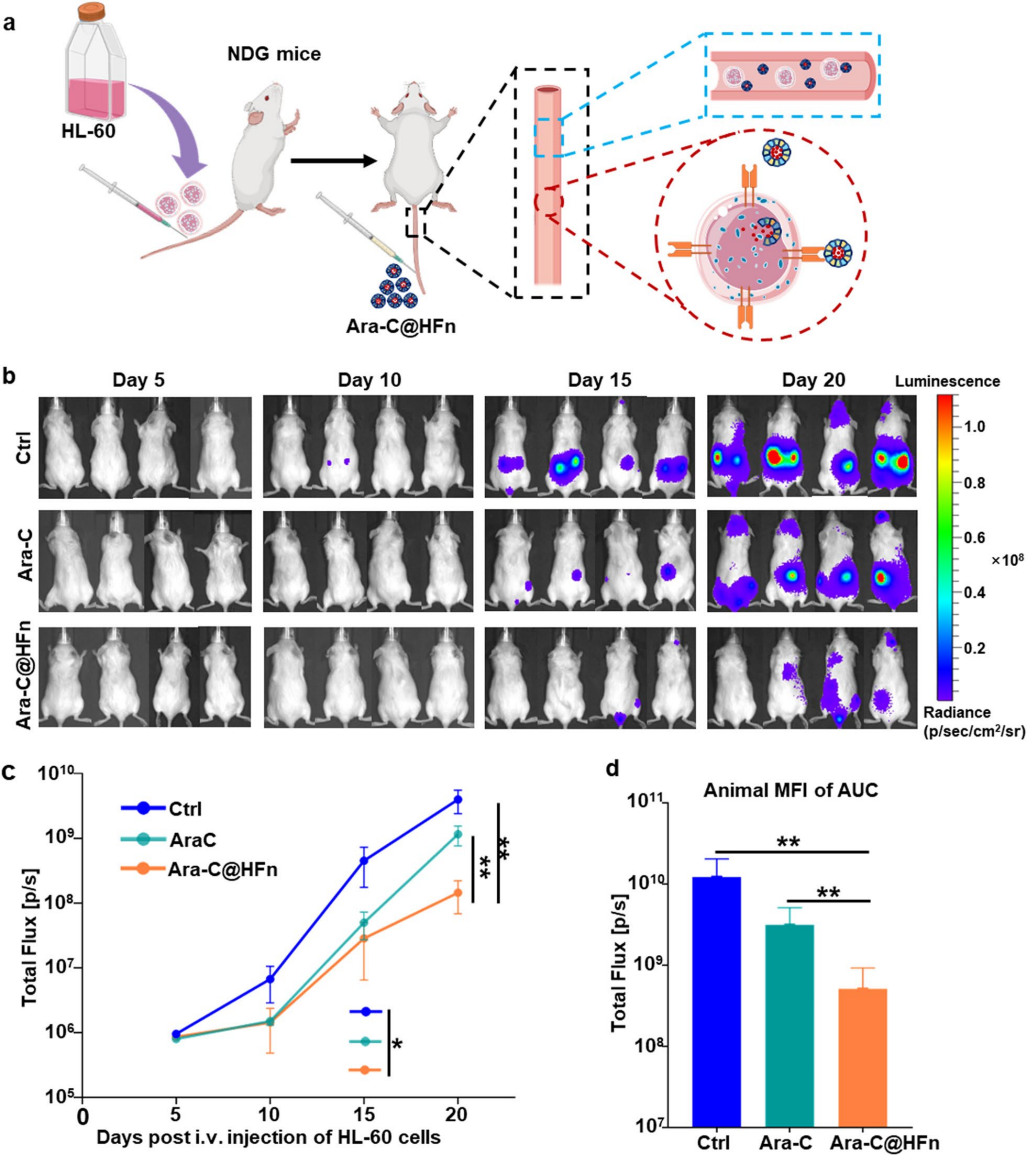
Anti-leukemia effects of Ara-C@HFn and free Ara-C in AML mouse models. The mice received six-round treatments of PBS, Ara-C (8 mg/kg) or Ara-C@HFn (with the same equivalent dose of Ara-C) (once every 2 days) after Luc-HL-60 cells were intravenous injected. **a** Illustration of Ara-C@HFn inhibiting AML cells in vivo. **b** Images of the leukemia burden from mice treated with PBS (Ctrl, n = 4), free Ara-C (n = 4) or Ara-C@HFn (n = 4) at different time points (Day 5, Day 10, Day 15 and Day 20), obtained using BLI. control (Ctrl, mice treated with PBS), free Ara-C and Ara-C@HFn. **c** Evolution of AML dissemination in mice among the three groups by measuring mouse BLI by IVIS Spectrum (n = 4). Data are presented as the mean of total flux [p/s] ± SE. *p < 0.05; **p < 0.01, Student‑Newman‑Keuls test. **d** Cumulative BLI in mice during the treatment quantified using the AUC calculated during assessments of AML evolution in each mouse among the three groups (n = 4). Data are presented as the mean AUC in total flux units [p/s] ± SE. **, p < 0.01, Student‑Newman‑Keuls test. *BLI* bioluminescent imaging, *AUC* area under the curve, *SE* standard error

The above series of results inspired us to further evaluate the suppression of leukemia in vivo. Since AML cells exhibit high expression levels of the TFRC, we hypothesized that Ara-C would be delivered into AML cells with higher efficiency through Ara-C@HFn to inhibit AML cell development (Fig. 3a) more efficiently. To evaluate the antineoplastic effect of free Ara-C and Ara-C@HFn, we randomly divided the Luc-HL-60 cell-bearing mice into three groups and treated them with PBS (Ctrl), free Ara-C or Ara-C@HFn.

AML disease progression was monitored throughout the experiment, and the bioluminescence signals in each mouse were analyzed with the IVIS spectrum system. As shown in (Fig. 3b, c), on Day 5, the bioluminescence signals were almost the same among the three groups of mice. With the progression of AML and the continuous administration of treatment, on the 10th day (after three administrations), the mice in the free Ara-C and Ara-C@HFn groups showed weaker bioluminescence signals than those in the Ctrl group. On the 15th day, mice in the Ara-C@HFn group showed the weakest bioluminescence signal, which was significantly lower than that in the Ctrl group. At the end of the experiment (20 days), mice in the Ara-C@HFn group still showed the weakest bioluminescence signal, which was significantly weaker than that in the Ctrl or Ara-C group. In addition, in a more global study of bioluminescence signals over time, which was performed by analyzing the AUC achieved during the analysis of the different treatments, Ara-C@HFn induced a significant blockade of AML progression by reducing the emitted luminescence by approximately 96% the total flux value observed in the Ctrl group, and Ara-C@HFn treatment resulted in an approximately 84% lower total flux than free Ara-C treatment (Fig. 3d). These results supported the idea that Ara-C@HFn treatment substantially outperformed Ara-C treatment, which was associated with low bioluminescence intensity levels indicating a reduction in the leukemia burden.

**Fig. 4:**
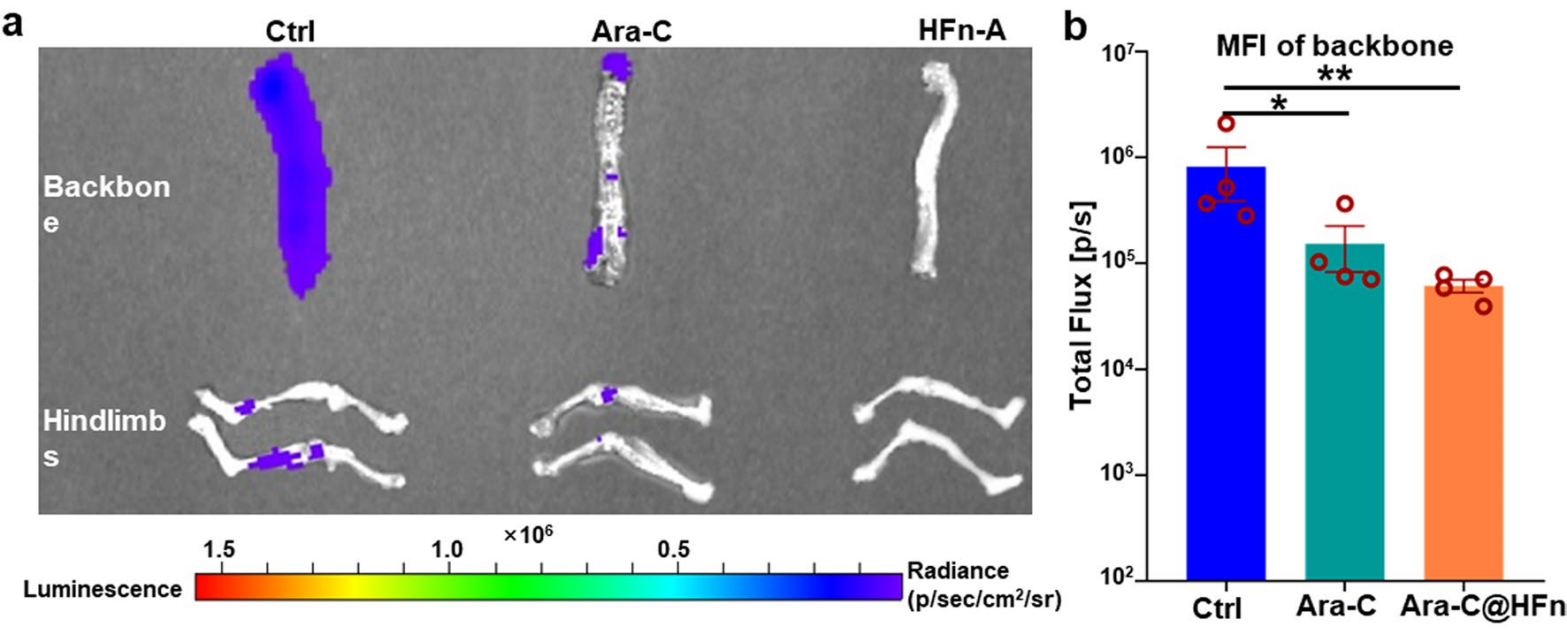
Anti-leukemia effect in AML cells affected organs ex vivo after mice were treated with Ara-C@HFn and free Ara-C. **a** Representative images of bone (backbone and hindlimbs) from mice among three groups. **b** The quantitation analysis of BLI in backbone from mice among three groups (n = 4), which were presented as mean of average radiance ± SE. Student‑Newman‑Keuls test was used for multiple comparison among three groups, and the significant difference between groups are indicated by * or ** when p-value was < 0.05 or < 0.01. *BLI* bioluminescent imaging, *SE* standard error

As some animals had signs of advanced disease at 20 days, mice in these three groups were euthanized, and local AML dissemination in bone marrow was studied by analyzing bioluminescence signals ex vivo using the IVIS spectrum. The results of fluorescence imaging showed that the backbone and hindlimbs in the Ara-C@HFn group had a much lower leukemia burden than those in the Ctrl group (Fig. 4a). In addition, to quantitatively compare the results of fluorescence imaging, we performed quantitative analysis on the bioluminescence signals of the backbone (Fig. 4b). The results showed that the backbone of mice treated with free Ara-C had a significantly lower leukemia burden than those in the Ctrl group, whereas the leukemia burden in the Ara-C@HFn group was consistently the lowest.

**Fig. 5:**
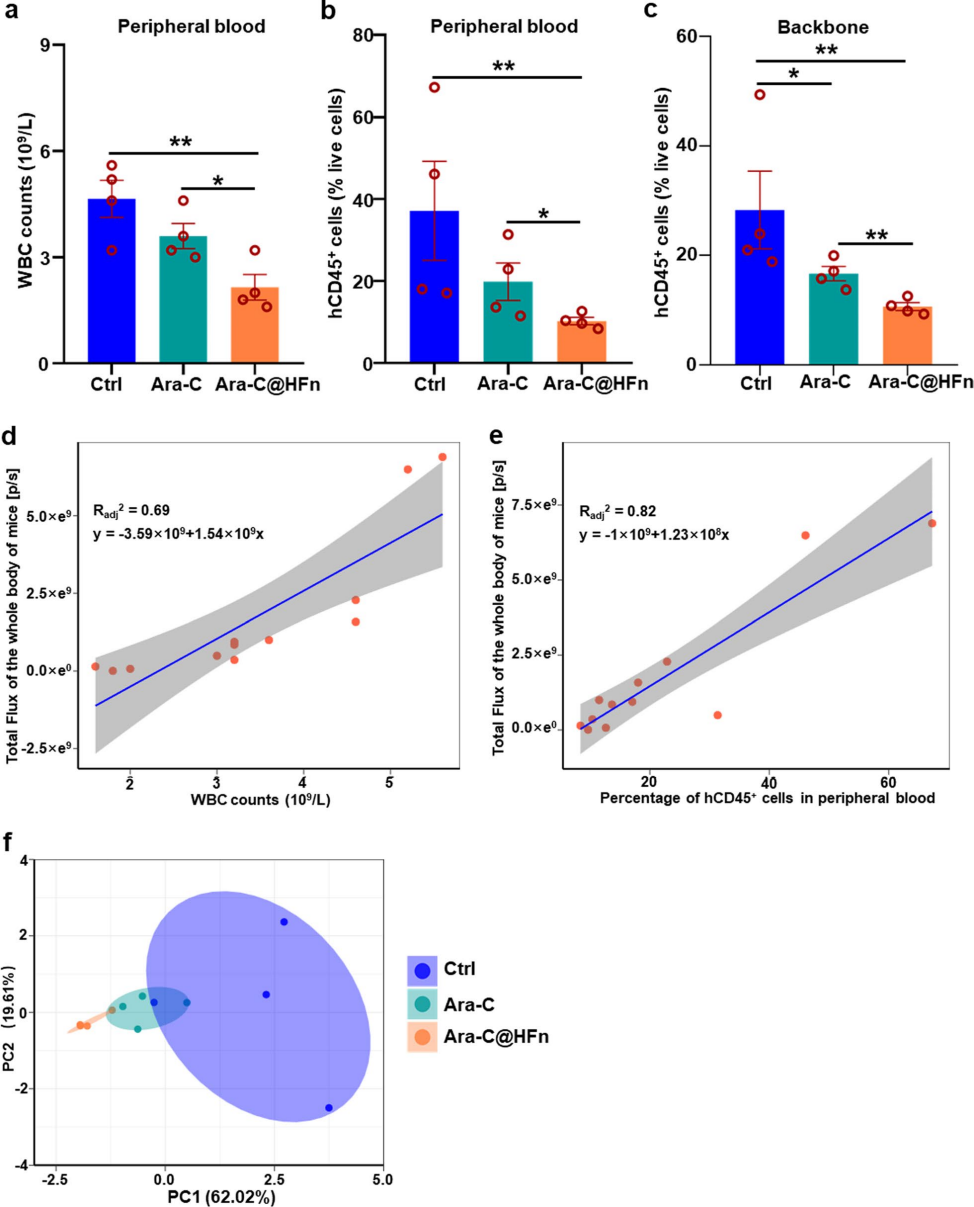
AML cell burden in backbone and peripheral blood and its correlation with the BLI in mice at the experimental terminal point. **a** WBC counts in peripheral blood from mice among the three treated groups (n = 4). Data are presented as the mean of counts ± SE. *p < 0.05; **p < 0.01, Student‑Newman‑Keuls test. **b**, **c** Flow cytometry analysis of the percentage of leukemia cells (hCD45^+^ cells) in peripheral blood and backbone from leukemia-bearing mice among the three treated groups (n = 4). Data are presented as the mean percentage of leukemia cells ± SE. *p < 0.05; **p < 0.01, Student‑Newman‑Keuls test. **d**, **e** Correlation analysis between the counts of WBCs in peripheral blood or the proportion of leukemic cells (hCD45^+^ cells) and the measurement of BLI in the whole body of mice at the experimental terminal point on Day 20. **f** A PCA plot was used to visualize the therapeutic index of mice among the three groups (based on the data of (**a**, **b**, **c**); the measurement of BLI in the lung, backbone, and whole body at the experimental terminal point), and each point represents one mouse. The ellipses show the sample distribution in each group at the 68% confidence level assuming a multivariate normal distribution. *BLI* bioluminescent imaging, *SE* standard error, *PCA* principal component analysis

We also analyzed the counts of WBCs in the peripheral blood because elevated WBC counts are a common indication of AML. The results showed that the counts of WBCs in the peripheral blood in mice treated with Ara-C@HFn were significantly lower than those in the Ctrl group and free Ara-C group (Fig. 5a). In addition, the flow cytometry analysis of peripheral blood (after RBCs were removed) and backbone (spinal cord cells) also suggested that the mice treated with Ara-C@HFn had a lower burden of AML cells (hCD45^+^ cells) (Fig. 5b, c). Incidentally, consistent with the results obtained from GEO data, the flow cytometry analysis also showed that the TFRC expression level in AML cells in peripheral blood did not decrease posttreatment (Additional file 1: Fig. S3c). In further assessments of the correlation between the above data, the results showed that the counts of WBCs and the proportion of hCD45^+^ cells in peripheral blood at the end of the experiment were positively correlated with the bioluminescence signals in all three groups of mice (Fig. 5d, e). To provide a clearer comparison among the three groups, we next generated a PCA plot based on the above data. As shown in Fig. 5f, the mice in the Ara-C@HFn group and the mice in the Ctrl group formed two clusters without intersection, indicating that the therapeutic indices (including the proportion of hCD45^+^ cells in peripheral blood, the bioluminescence signals of whole body and local organs, etc.) in the two groups of mice were significantly different, which again suggested that Ara-C@HFn induced a blockade of AML progression.

In general, the above results indicate that Ara-C@HFn has a better AML blockade effect than free Ara-C.

### Acute toxicity assessment of Ara-C@HFn

Although many previous studies have reported that HFn has good biosafety in vivo [17, 27, 30, 31], we once again evaluated the acute toxicity (48 h post treatment) of HFn in mice by treating them with Ara-C@HFn. As expected, Ara-C@HFn, as well as the free Ara-C, caused a decrease in the counts of WBCs and platelets (Additional file 1: Fig. S4a, b), while there was no difference in the counts of RBCs and hemoglobin concentrations between the Ara-C@HFn group and the PBS group (Additional file 1: Fig. S4c, d). According to pharmacological principles and corresponding detection from mice treated with free Ara-C, the decrease of WBCs and platelet counts in mice treated with Ara-C@HFn should be caused by Ara-C. Moreover, Ara-C@HFn did not cause any damage to the major organs in mice, such as the heart, liver, spleen, lung, and kidney, while the free Ara-C caused slight changes in the ratio of red pulp to white pulp of spleen (Additional file 1: Fig. S4e). These results again indicated that HFn has good biocompatibility and confirmed the good biosafety of HFn for drug delivery.

### Prospects of HFn for therapeutic applications in AML

**Fig. 6:**
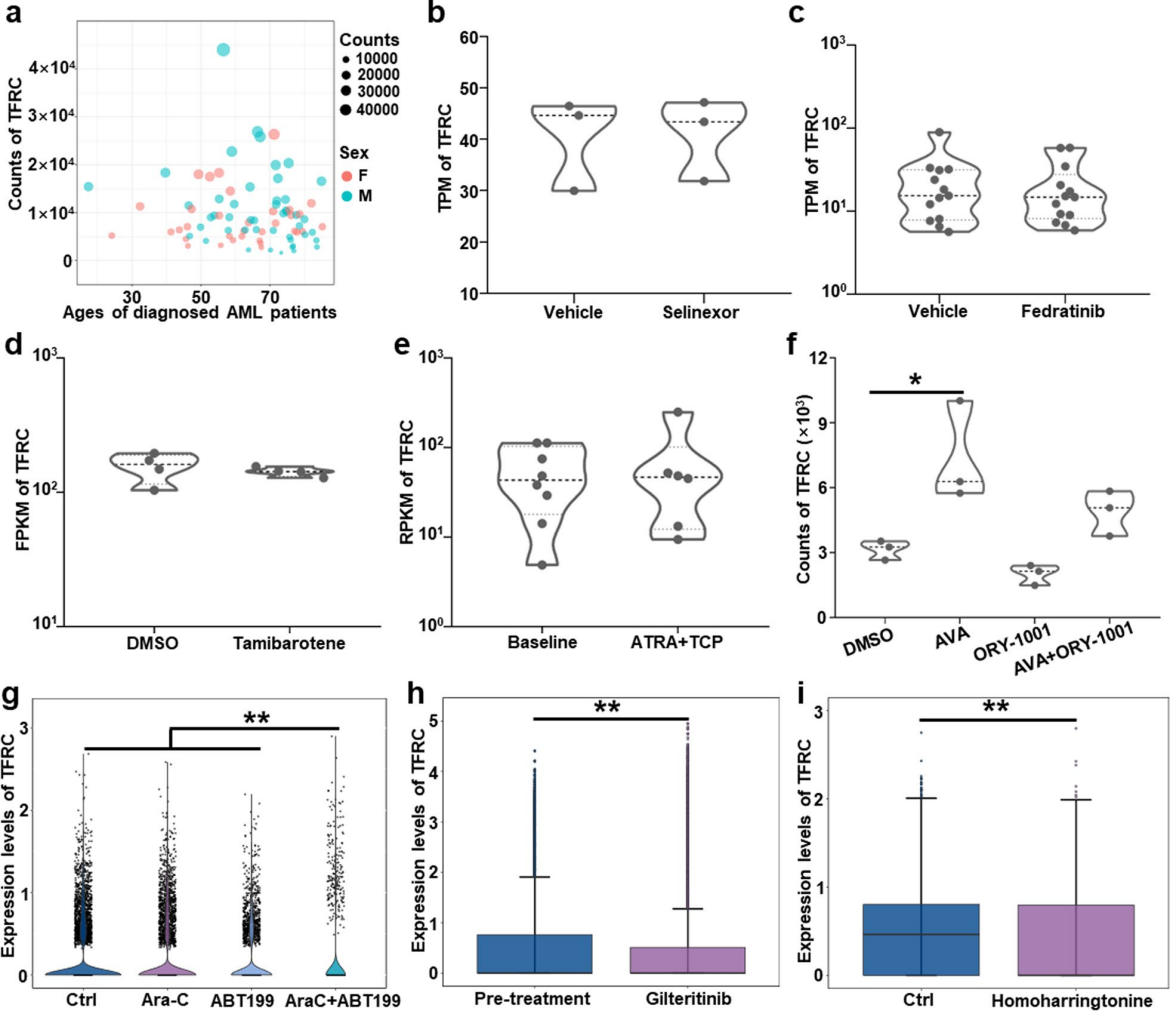
Characterization of TFRC gene expression in AML cells isolated from AML patients. **a** The expression levels of TFRC in AML cells isolated from AML patients of various ages and sexes (GSE165656, n = 81). **b**, **c**, **d** The TPM or FPKM changes in TFRC gene expression in AML cells based on RNA-seq data after treatment with selinexor (GSE199454, n = 3), fedratinib (GSE199456, n = 13) or tamibarotene (GSE155559, n = 4). **e** RPKM changes in TFRC gene expression in AML cells based on RNA-seq data after treatment with ATRA + TCP (administration combined with ATRA and TCP, GSE151594, n = 8 in baseline and n = 6 in ATRA + TCP). **f** The changes in TFRC expression in AML cells based on RNA-seq data after treatment with AVA, ORY-1001 and AVA + ORY-1001 (GSE182150, n = 3). *p < 0.05, Student‑Newman‑Keuls test. **g** The changes in the expression levels of TFRC in AML cells based on scRNA-seq data after treatment with Ara-C, ABT199 and Ara-C + ABT199 (GSE178910, n = 1). **p < 0.01, Student‑Newman‑Keuls test. **h**, **i** The changes in the expression levels of TFRC in AML cells based on scRNA-seq data after treatment with gilteritinib (GSE199333, n = 7 and n = 13 in pre- and post-treated groups) and homoharringtonine (GSE182020, n = 1). **p < 0.01, t test. *TPM* transcripts per million; *FPKM* fragment counts per kilobase of transcript sequence per million base pairs sequenced; *RPKM* reads per kilobase of exon per million reads mapped, *ATRA* all-trans retinoic acid, *TCP* tranylcypromine; *AVA* Avapritinib; *scRNA-seq* single-cell RNA sequencing

The above results confirmed our hypothesis based on the analysis of data obtained from the GEO database; that is, the expression of TFRC in AML cells treated with Ara-C was not significantly decreased, which enables the effective inhibition of the development of AML cells by Ara-C@HFn. This result also motivated us to analyze the prospect of applying HFn nanocages by loading drugs for AML treatment based on the relevant data from the GEO database. Initially, we analyzed the clinical data and RNA sequencing data obtained from 81 AML patients (GSE165656) and found that there was no significant correlation (positive or negative correlation) between the sex or age of diagnosed AML patients and the TFRC expression level in AML cells (Fig. 6a). Moreover, we found that the expression level of TFRC in AML patient-derived leukemia cells did not show a significant difference from AML cells with the different expression levels of GPR56 or CD34 (GSE129094, Additional file 1: Fig. S5a).

Next, we analyzed the changes in TFRC expression in AML cells after treatment with several common drugs that have been used for the clinical treatment of AML. The results showed that the expression level of TFRC did not change in AML cells after treatment with selinexor (GSE199454, Fig. 6b), fedratinib (GSE199456, Fig. 6c), Tamibarotene (GSE155559, Fig. 6d), ATRA + TCP (combined with the LSD1 inhibitor tranylcypromine with all-trans retinoic acid, GSE151594, Fig. 6e), ORY-1001 (GSE182150, Fig. 6f), Ara-C or ABT199 (GSE178910, Fig. 6g). The expression level of TFRC was increased when AML cells were treated with AVA (Avapritinib, Fig. 6f) or Ara-C + ABT199 (Ara-C combined with ABT199, Fig. 6g), while the expression level of TFRC was decreased when AML cells were treated with gilteritinib (GSE199333, Fig. 6h) or homoharringtonine (GSE182020, Fig. 6i).

Additionally, to better guide the development of HFn nanocages as drug-targeted delivery systems for AML therapy, we analyzed the expression of TFRC in AML cells in many clinical scenarios using the data obtained from the GEO database. Our results suggest that although the TFRC expression levels in newly diagnosed and relapsed AML patients were consistent overall (GSE156008, GSE199451 and GSE153348; Additional file 1: Fig. S5b–d), the TFRC expression levels in AML cells in relapsed patients tended to increase (Additional file 1: Fig. S5c, d). After treatment with the 7 + 3 regimens, there was no significant difference in TFRC expression in AML cells between the complete remission (CR) group and the noncomplete remission (Non-CR) group (GSE103424 and GSE164894; Additional file 1: Fig. S5e, f). In addition, the TFRC expression in AML cells did not change significantly in two other clinical scenarios, which included those who exhibited progression and those who did not among AML patients administered combined venetoclax and azacitidine treatment (GSE155431, Additional file 1: Fig. S5g) or the responders, partial responders and non-responders among AML patients treated with fedratinib (GSE199455, Additional file 1: Fig. S5h).

Since the constant expression of TFRC is a prerequisite for drug-targeted delivery based on HFn nanocages, the above results suggested that the drug-targeted delivery system based on HFn nanocages has the potential to improve the therapeutic effect of most of the analyzed drugs for various clinical scenarios, except during the use of the drugs gilteritinib and homoharringtonine.

## Discussion

Although several studies have reported that HFn could be used as a drug carrier for targeted drug delivery to cancer cells [15, 17, 19, 23], there are no reports on the differences in the expression levels of the HFn receptor TFRC in the target cells before and after treatment, which will have an important impact on the efficiency of drug delivery achieved by targeted drug delivery systems based on HFn nanocages. In addition, as a first-line chemotherapy drug for AML patients, Ara-C has a history of more than 40 years of use in the clinic [3], so further improving the suppression effect of Ara-C on AML cells or reducing its toxicity and side effects has direct and important clinical value. After confirming that the expression levels of TFRC from AML cells were not significantly downregulated after treatment with Ara-C, Ara-C@HFn was generated here to improve the antineoplastic effect of Ara-C in AML cells.

In fact, as a pyrimidine nucleoside analog, Ara-C has a short half-life in plasma, rapid inactivation in plasma, extensive hepatic metabolism and fast renal elimination [7, 32, 33], and a continuous intravenous infusion of Ara-C is necessary to achieve sustained and effective antineoplastic concentrations. Nevertheless, prolonged exposure to Ara-C at a high concentration induces severe adverse effects, including leukopenia and thrombocytopenia, gastrointestinal toxicity, hepatic dysfunction, and infectious complications [7, 32]. Some efforts have been made to increase the elimination half-life of free Ara-C, such as the development of CPX-351, which is a marketed liposome that encapsulates daunorubicin and Ara-C [34]. However, the liposome changes only the drug pharmacokinetics of Ara-C, rather than proving preferential uptake by leukemia cells. Thus, CPX-351 is associated with a low rate of clinical response in high-risk or relapsed AML patients and exhibits similar toxicity to free Ara-C during intensive chemotherapy. In mammals, extracellular ferritin (nanocages) that are self-assembled by 24 subunits can be found in serum in a secreted form [35], and HFn can be effectively delivered to tumor cells through TFRC-mediated endocytosis [36, 37]. Previous studies have confirmed that the serum half-life of drug-loaded HFn can be more than twice that of free Ara-C [19, 33]. Therefore, the analysis results that there was a constant expression levels of TFRC in AML cells encouraged us to generate the Ara-C@HFn in this study, which had the potential to reduce the adverse effects of free Ara-C on AML therapy and had a more potent antineoplastic effect in AML than free Ara-C, which was as expected.

In this study, the Ara-C@HFn showed a better antineoplastic effect in AML than free Ara-C in vitro and in vivo. Although the daily average dose of Ara-C used in mice was approximately one-sixth to one-third of that used in AML patients for induction therapy through Ara-C@HFn [38, 39], as well as much less than that used in other studies based on free Ara-C in mice [40–42], it significantly reduced the leukemia burden in mice. The conclusions obtained from the data based on the GEO database are consistent with our previous studies [17] (AML cells exhibited a high level of TFRC) and our results in this study (TFRC expression was not significantly downregulated after Ara-C treatment). Moreover, the data analyzed based on the database are not inconsistent with each other and, in fact, support each other, indicating that the conclusions obtained based on the data from the database have high reliability. In addition to focusing on Ara-C, we also performed a very detailed analysis of the prospect of the use of HFn in AML therapy (by analyzing the effect of other AML-related antineoplastic drugs on the expression level of TFRC) and analyzed the expression level of TFRC in AML cells under various clinical scenarios. Note that the results of cell cycle analysis suggested that Ara-C@HFn and free Ara-C exhibited similar S and G_2_/M arrest characteristics in AML cells, which might be due to Ara-C@HFn can improve the internalization speed of Ara-C without changing the work mechanism of Ara-C, and the cell cycle of cells treated with free Ara-C or Ara-C@HFn may have been consistent when detecting the cell cycle with the fact that each cell can fully contact Ara-C in vitro experiments. In general, these results could be a very important research basis for guiding the use of HFn nanocages during AML therapy.

Inevitably, there were some limitations to this study. For example, we did not explore the maximum amount of Ara-C loaded by HFn, mainly because we considered that an excessive amount of Ara-C loading might lead to a small number of injected HFn nanocages, which would have ultimately made it difficult to uniformly deliver Ara-C at levels that could effectively induce a suppression effect to each leukemia cell, thereby reducing the suppression ability of Ara-C@HFn in AML. However, the suppression effect of Ara-C@HFn when used with various molar ratios of HFn and Ara-C on AML will become part of our future research to promote the clinical application of Ara-C@HFn. In addition, although we have analyzed the expression of TFRC in AML cells under many different drug treatments and various clinical scenarios, due to the limited space, our analysis was not sufficiently comprehensive, and a more comprehensive analysis will also become an important area of study for us to further develop targeted drug delivery systems based on HFn nanocages in the future. Finally, we did not analyze the expression levels of TFRC in other tumor cells after treatment with related drugs, especially in solid tumors. Solid tumors have great heterogeneity and a complex tumor microenvironment [43, 44], and the analysis results may not be very optimistic. However, we believe that further analysis of relevant sequencing data from clinical samples in the future will be able to identify promising drugs suitable for HFn nanocages (to generate targeted drug delivery systems) in solid tumor therapy.

Additionally, since the clinical samples collected by each research team are limited, it is impossible to evaluate the therapeutic effects of all relevant clinical drugs (such as the therapy drugs for AML) in all relevant clinical scenarios. The results of this study suggest that using existing open-source public databases, such as GEO and The Cancer Genome Atlas (TCGA), to summarize the relevant data can also provide useful guidance for research designs, which will greatly accelerate the process of future drug development. We believe that this research method of mining and sorting data from public databases as used in this study is expected to accelerate the process through which we develop antitumor drugs.

## Conclusion

In conclusion, after determining that the expression of TFRC in AML cells from AML patients did not significantly decrease after treatment with Ara-C, we generated Ara-C@HFn, which can be used to deliver Ara-C to AML cells specifically by recognizing TFRC that is expressed on AML cells at a high level. Ara-C@HFn had significantly higher suppression efficiency in AML cells than free Ara-C in vitro or in vivo, and effectively reduced the leukemia burden in AML mice. Therefore, Ara-C@HFn is very promising for clinical application in the future, as it has good biocompatibility and an excellent antineoplastic effect in AML cells, as observed in this study. Furthermore, by analyzing the sequencing data obtained from clinical samples from the GEO database, we identified a variety of related drugs that did not cause significant downregulation of TFRC expression in AML cells, as well as 2 individual drugs and 1 combination drug that caused significant downregulation of TFRC expression in AML cells, which provided important reference data for our further efforts to develop a targeted drug delivery system based on HFn.

## Supplementary Information

**Additional file 1:** **Fig. S1** Characterization of TFRC gene expression in cells obtained from different sources. **a** The expression levels of TFRC in bone marrow endothelial progenitor cells isolated from AML patients or healthy donors (GSE197907, n=3). *p<0.05, t test. **b** The expression levels of TFRC in bone marrow immune cells isolated from AML patients or healthy donors (GSE154109, n=4 and n=8 in healthy donors and AML patients). *p<0.05, t test. **c** TPM changes in TFRC gene expression in AML cells based on RNA-seq data after treatment with Ara-C (GSE145061, n=3). *p<0.05, t test. **d** FPKM changes in TFRC gene expression in AML cells based on RNA-seq data after treatment with Ara-C (GSE146592, n=6 on Day 0, n=4 on Day 8, and n=5 on Day 29). *p<0.05, Student‑Newman‑Keuls test. TPM, transcripts per million; FPKM, fragment counts per kilobase of transcript sequence per million base pairs sequenced. **Fig. S2 **Assessments of intracellular internalization and cytotoxicity of Ara-C@HFn on HL-60 cells blocking with anti-TFRC antibodies. **a**, **b** Representative confocal laser scanning microscope images of Ara-C@HFn (Cy5 labeled) endocytosed by normal HL-60 **a** and anti-TFRC antibody blocked HL-60 **b** cells (green, lysosome; blue, nucleus; red, Cy5–Ara-C@HFn; scale bar, 5 μm). **c** Relative viability analysis of normal HL-60 and anti-TFRC antibody blocked HL-60 cells after treatment with different concentrations of Ara-C@HFn for 48 h (n=4). **d** The kinetics of Ara-C release from Ara-C@HFn in vitro at pH 5.0 and pH 7.0 at 37 ℃ (n=3); **e** Percentage analysis of the G0/G1, S and G2/M phases of the cell cycle in HL-60 cells treated with free Ara-C or Ara-C@HFn (n=3). **f** Ratio analysis of the percentage of S phase and G0/G1 phase of the cell cycle in HL-60 cells after treatment with free Ara-C or Ara-C@HFn (n=3). *p<0.05, Student‑Newman‑Keuls test. **Fig. S3** Assessments of Ara-C@HFn targeting ability in leukemia cells *in vivo* and expression characteristics of TFRC in AML cells after Ara-C treatment. **a** Flow cytometry analysis of Cy5-Ara-C@HFn binding to cells extracted from the peripheral blood, hindlimbs or spleen of leukemia-bearing mice (n=3). **b** Percentage of hCD45^+^ cells from HFn^+^ cells extracted from the peripheral blood, hindlimbs or spleen of leukemia-bearing mice (n=3). **c** TFRC expression in AML cells (hCD45^+^ cells) in peripheral blood obtained from mice in the three treatment groups (n=4). **Fig. S4** Biosafety evaluation of Ara-C@HFn. **a**–**c** WBC, PLT and RBC counts in peripheral blood obtained from mice treated with PBS, free Ara-C or Ara-C@HFn (n=3). *p<0.05; **p<0.01; Student‑Newman‑Keuls test. **d** The concentration of HGB in peripheral blood obtained from mice treated with PBS, free Ara-C or Ara-C@HFn (n=3). **e** Histopathological analysis of tissue sections from mice treated with PBS, free Ara-C or Ara-C@HFn (n=3). **Fig. S5** Characteristics of TFRC expression in AML patient-derived leukemia cells in various clinical scenarios. **a** TPM expression of TFRC in AML cells with different expression levels of GPR56 or CD34 based on RNA-seq data (GSE129094, n=7 in the GPR56^−^ CD34^−^ group, n=7 in the GPR56^+^ CD34^-^ group, n=6 in the GPR56^+^ CD34^+^ group). **b**–**d** The TFRC levels in AML cells from newly diagnosed and relapsed AML patients **b**, GSE156008, n=9 in the newly diagnosed group and n=15 in the relapsed group; **c**, GSE199451, n=11; d, GSE153348, n=6). **e**, **f** The TFRC levels in AML cells from the CR group and non-CR group (**e**), GSE103424, n=18; **f** GSE164894, n=15 in the CR group and n=9 in the non-CR group). (g) The TFRC levels in AML cells obtained from progressor and non-progressor groups among AML patients administered combined venetoclax and azacitidine treatment (GSE155431, n=6 in the non-progressor group and n=3 in the progressor group). **h** TPM expression of TFRC in AML cells of non-responder, partial responder and responder groups among AML patients treated with fedratinib (GSE199455, n=3, n=4, and n=8, respectively). *TPM* transcripts per million, *CR* complete remission, *non-CR* noncomplete remission.

## Data Availability

GEO data was downloaded from the GEO database (http://www.ncbi.nlm.nih.gov/projects/geo/). The data supporting the findings of this study is available from the corresponding authors on reasonable request.

## Abbreviations

Ara-C: Cytarabine arabinoside
AML: Acute myeloid leukemia
TFRC: Transferrin receptor 1
HFn: Heavy ferritin chain
TFRs: Transferrin receptors
SCID: Severe combined immune deficiency
SPF: Specific pathogen‑free
HPLC: High-performance liquid chromatography
TEM: Transmission electron microscopy
SDS‒PAGE: SDS–polyacrylamide gel electrophoresis
scRNA-seq: Single-cell RNA sequencing
PCA: Principal component analysis
