# Supplementary material for: Expression of TFRC helps to improve the antineoplastic effect of Ara-C on AML cells through a targeted delivery carrier
Source: J Nanobiotechnology. 2023 Apr 11;21:126. doi: 10.1186/s12951-023-01881-8 (PMC10088114; doi:10.1186/s12951-023-01881-8)
Supplement: Supplementary file 1 — Additional file 1: Fig. S1 Characterization of TFRC gene expression in cells obtained from different sources. a The expression levels of TFRC in bone marrow endothelial progenitor cells isolated from AML patients or healthy donors (GSE197907, n=3). *p<0.05, t test. b The expression levels of TFRC in bone marrow immune cells isolated from AML patients or healthy donors (GSE154109, n=4 and n=8 in healthy donors and AML patients). *p<0.05, t test. c TPM changes in TFRC gene expression in AML cells based on RNA-seq data after treatment with Ara-C (GSE145061, n=3). *p<0.05, t test. d FPKM changes in TFRC gene expression in AML cells based on RNA-seq data after treatment with Ara-C (GSE146592, n=6 on Day 0, n=4 on Day 8, and n=5 on Day 29). *p<0.05, Student‑Newman‑Keuls test. TPM, transcripts per million; FPKM, fragment counts per kilobase of transcript sequence per million base pairs sequenced. Fig. S2 Assessments of intracellular internalization and cytotoxicity of Ara-C@HFn on HL-60 cells blocking with anti-TFRC antibodies. a, b Representative confocal laser scanning microscope images of Ara-C@HFn (Cy5 labeled) endocytosed by normal HL-60 a and anti-TFRC antibody blocked HL-60 b cells (green, lysosome; blue, nucleus; red, Cy5–Ara-C@HFn; scale bar, 5 μm). c Relative viability analysis of normal HL-60 and anti-TFRC antibody blocked HL-60 cells after treatment with different concentrations of Ara-C@HFn for 48 h (n=4). d The kinetics of Ara-C release from Ara-C@HFn in vitro at pH 5.0 and pH 7.0 at 37 ℃ (n=3); e Percentage analysis of the G0/G1, S and G2/M phases of the cell cycle in HL-60 cells treated with free Ara-C or Ara-C@HFn (n=3). f Ratio analysis of the percentage of S phase and G0/G1 phase of the cell cycle in HL-60 cells after treatment with free Ara-C or Ara-C@HFn (n=3). *p<0.05, Student‑Newman‑Keuls test. Fig. S3 Assessments of Ara-C@HFn targeting ability in leukemia cells in vivo and expression characteristics of TFRC in AML cells after Ara-C treatment. a Flow [file 12951_2023_1881_MOESM1_ESM.docx]

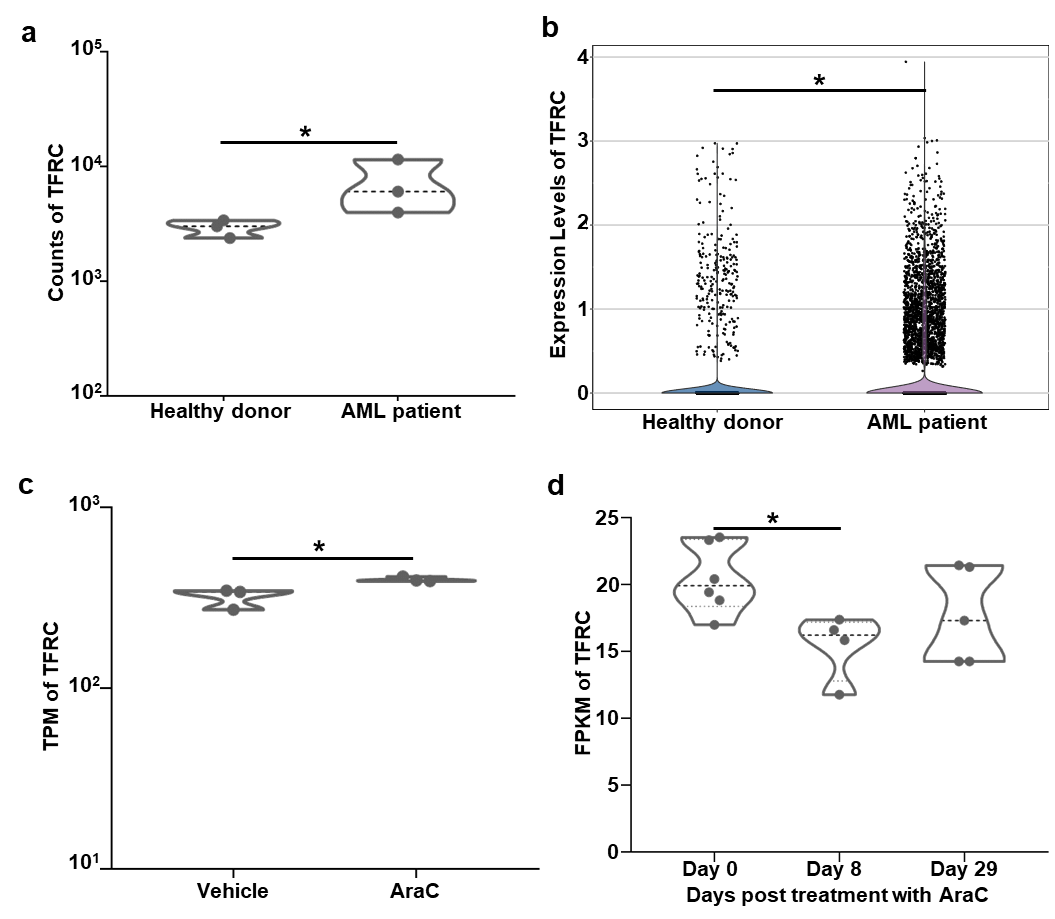


**Fig. S1** Characterization of TFRC gene expression in cells obtained from different sources. (a) The expression levels of TFRC in bone marrow endothelial progenitor cells isolated from AML patients or healthy donors (GSE197907, n=3). *, p<0.05, t test. (b) The expression levels of TFRC in bone marrow immune cells isolated from AML patients or healthy donors (GSE154109, n=4 and n=8 in healthy donors and AML patients). *, p<0.05, t test. (c) TPM changes in TFRC gene expression in AML cells based on RNA-seq data after treatment with Ara-C (GSE145061, n=3). *, p<0.05, t test. (d) FPKM changes in TFRC gene expression in AML cells based on RNA-seq data after treatment with Ara-C (GSE146592, n=6 on Day 0, n=4 on Day 8, and n=5 on Day 29). *, p<0.05, Student‑Newman‑Keuls test. TPM, transcripts per million; FPKM, fragment counts per kilobase of transcript sequence per million base pairs sequenced.


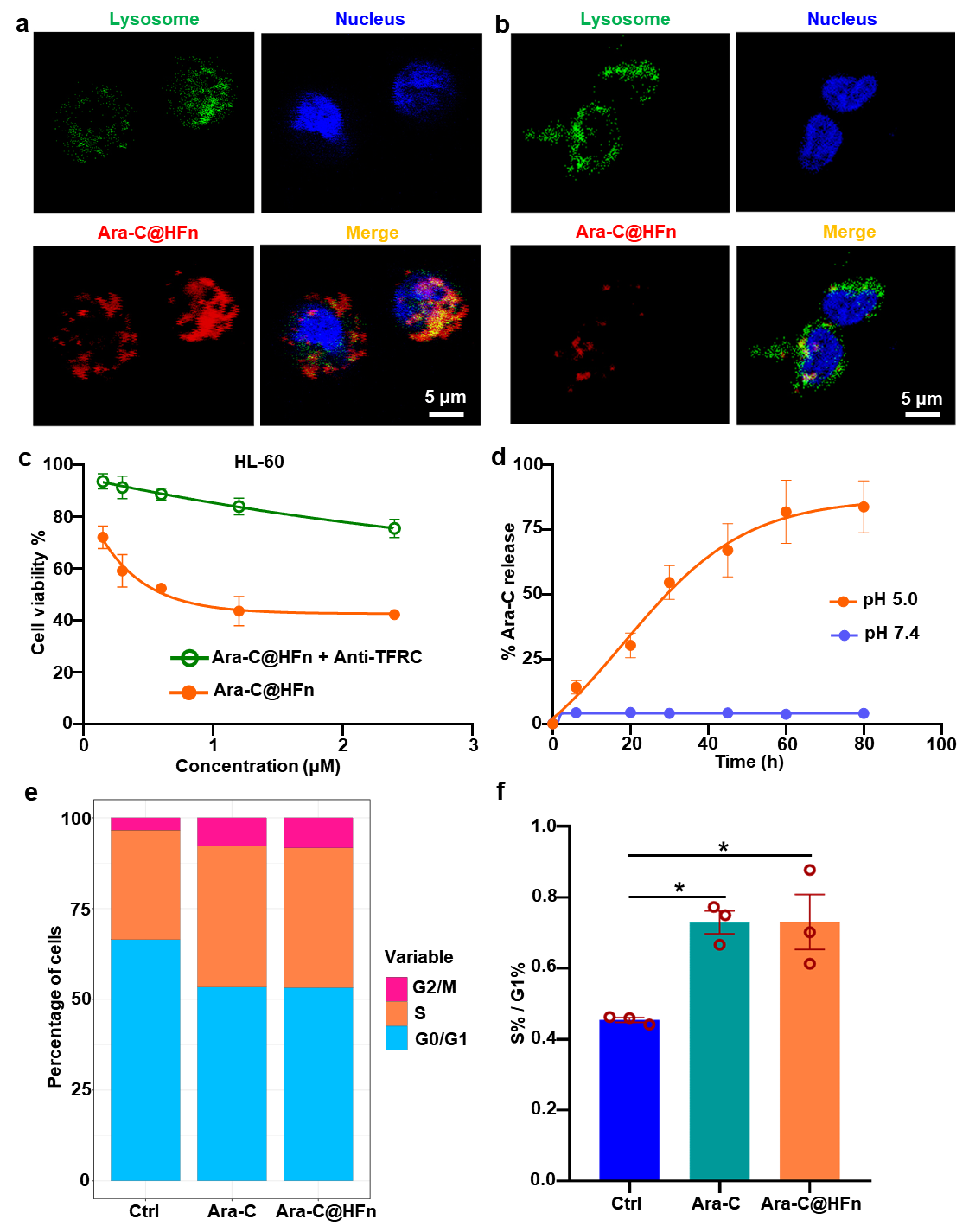


**Fig. S2** Assessments of intracellular internalization and cytotoxicity of Ara-C@HFn on HL-60 cells blocking with anti-TFRC antibodies. (a, b) Representative confocal laser scanning microscope images of Ara-C@HFn (Cy5 labeled) endocytosed by normal HL-60 (a) and anti-TFRC antibody blocked HL-60 (b) cells (green, lysosome; blue, nucleus; red, Cy5–Ara-C@HFn; scale bar, 5 μm). (c) Relative viability analysis of normal HL-60 and anti-TFRC antibody blocked HL-60 cells after treatment with different concentrations of Ara-C@HFn for 48 h (n = 4). (d) The kinetics of Ara-C release from Ara-C@HFn *in vitro* at pH 5.0 and pH 7.0 at 37 ℃ (n = 3); (e) Percentage analysis of the G0/G1, S and G2/M phases of the cell cycle in HL-60 cells treated with free Ara-C or Ara-C@HFn (n = 3). (f) Ratio analysis of the percentage of S phase and G0/G1 phase of the cell cycle in HL-60 cells after treatment with free Ara-C or Ara-C@HFn (n = 3). *, p<0.05, Student‑Newman‑Keuls test.


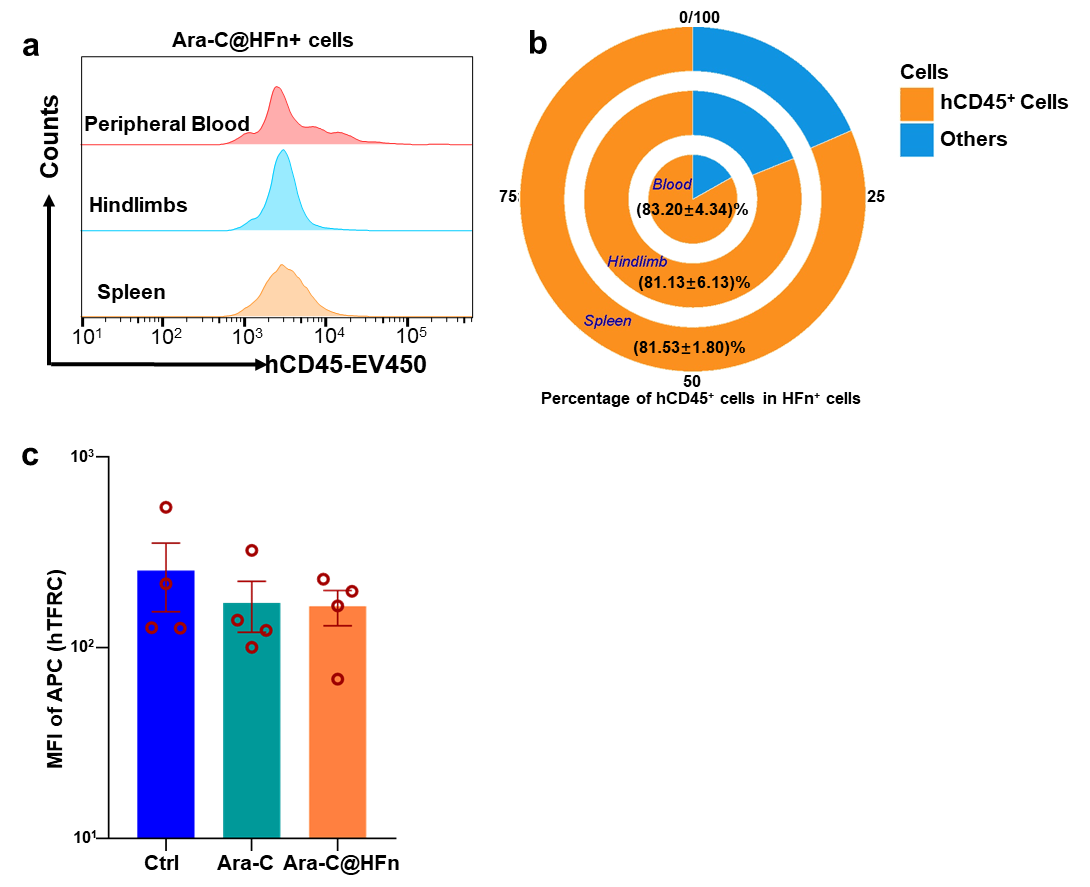


**Fig. S3** Assessments of Ara-C@HFn targeting ability in leukemia cells *in vivo* and expression characteristics of TFRC in AML cells after Ara-C treatment. (a) Flow cytometry analysis of Cy5-Ara-C@HFn binding to cells extracted from the peripheral blood, hindlimbs or spleen of leukemia-bearing mice (n=3). (b) Percentage of hCD45^+^ cells from HFn^+^ cells extracted from the peripheral blood, hindlimbs or spleen of leukemia-bearing mice (n=3). (c) TFRC expression in AML cells (hCD45^+^ cells) in peripheral blood obtained from mice in the three treatment groups (n=4).


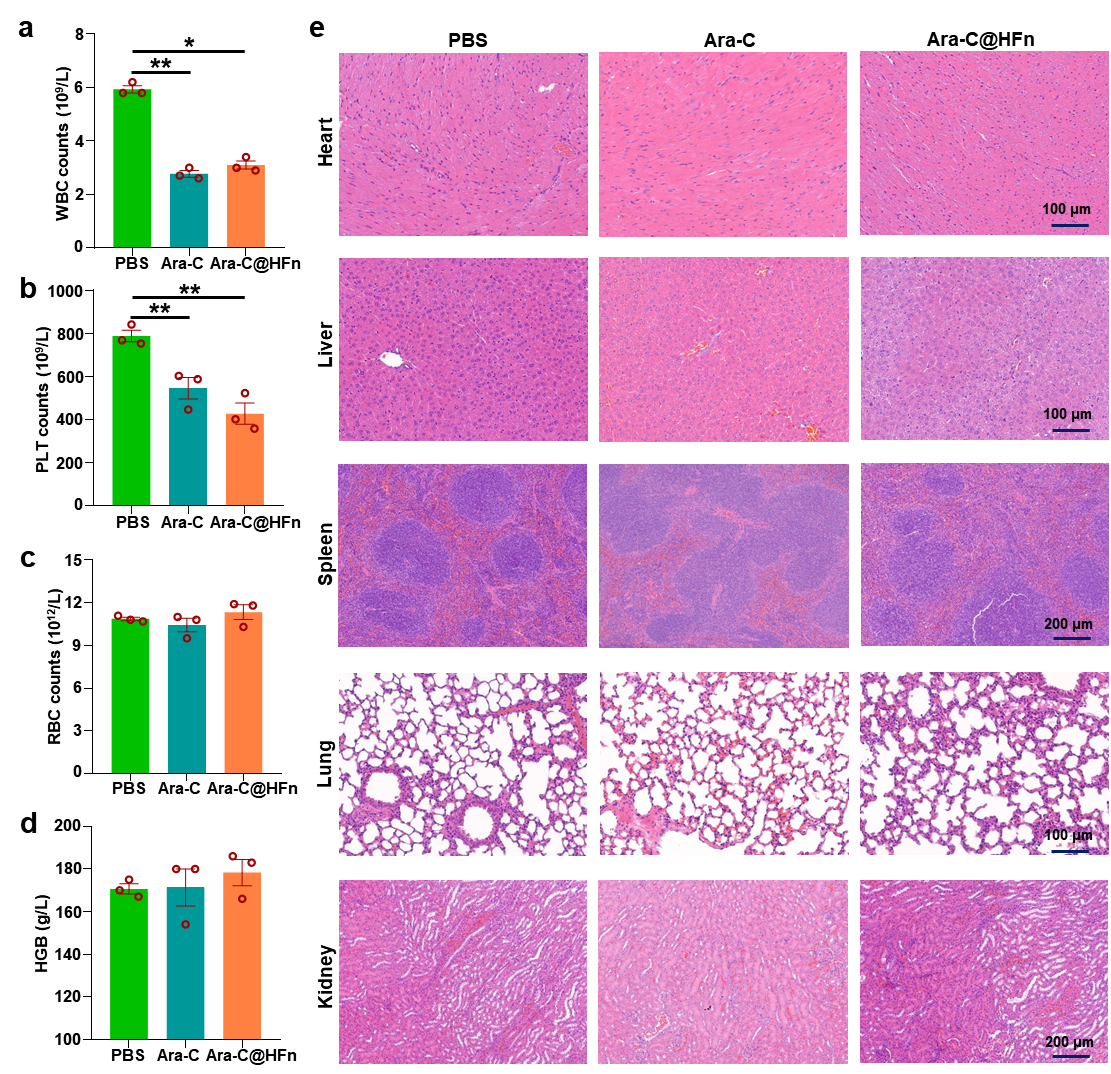


**Fig. S4** Biosafety evaluation of Ara-C@HFn. (a, b, c) WBC, PLT and RBC counts in peripheral blood obtained from mice treated with PBS, free Ara-C or Ara-C@HFn (n=3). *, p<0.05; **, p<0.01; Student‑Newman‑Keuls test. (d) The concentration of HGB in peripheral blood obtained from mice treated with PBS, free Ara-C or Ara-C@HFn (n=3). (e) Histopathological analysis of tissue sections from mice treated with PBS, free Ara-C or Ara-C@HFn (n=3).


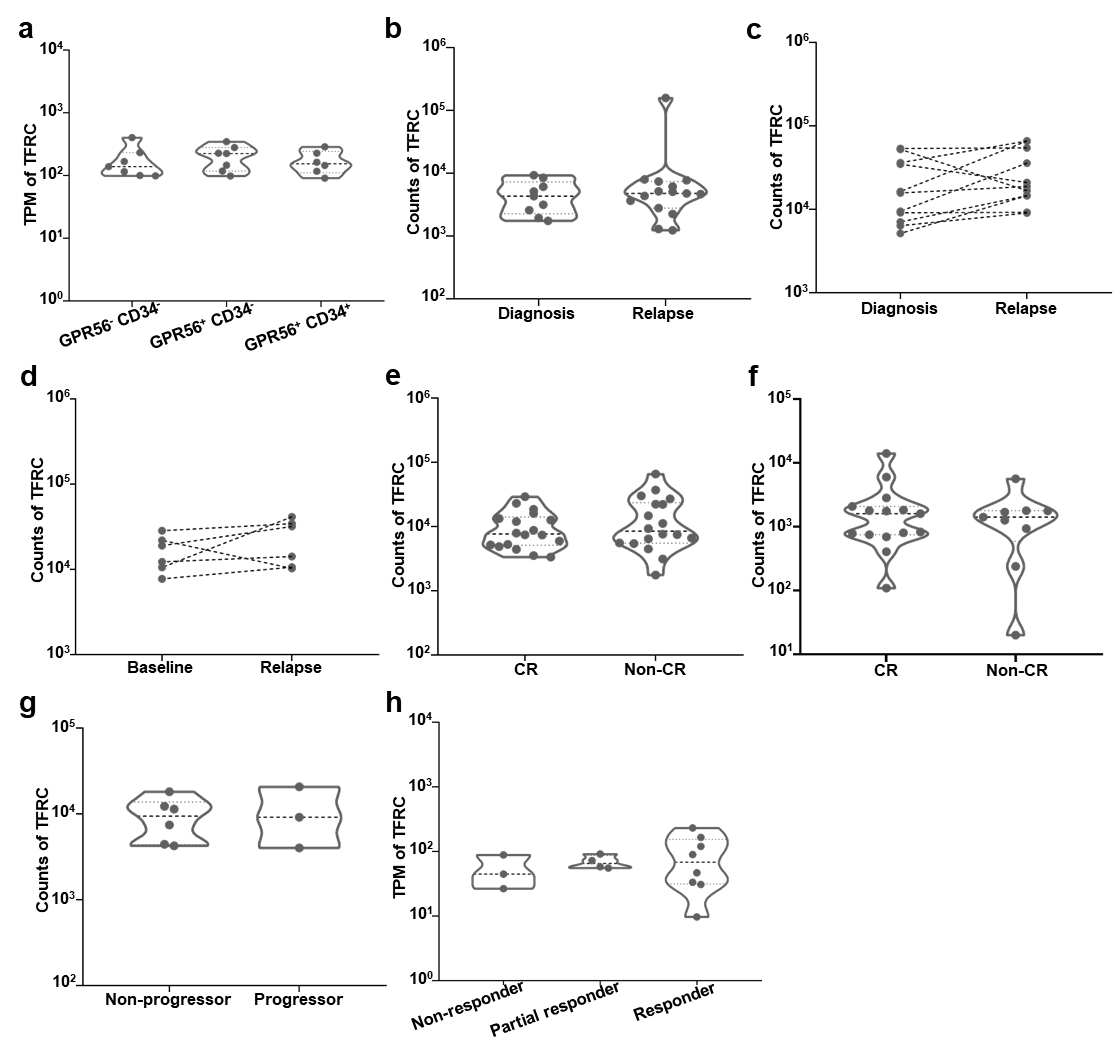


**Fig. S5** Characteristics of TFRC expression in AML patient-derived leukemia cells in various clinical scenarios. (a) TPM expression of TFRC in AML cells with different expression levels of GPR56 or CD34 based on RNA-seq data (GSE129094, n=7 in the GPR56^-^ CD34^-^ group, n=7 in the GPR56^+^ CD34^-^ group, n=6 in the GPR56^+^ CD34^+^ group). (b, c, d) The TFRC levels in AML cells from newly diagnosed and relapsed AML patients (b, GSE156008, n=9 in the newly diagnosed group and n=15 in the relapsed group; c, GSE199451, n=11; d, GSE153348, n=6). (e, f) The TFRC levels in AML cells from the CR group and non-CR group (e, GSE103424, n=18; f, GSE164894, n=15 in the CR group and n=9 in the non-CR group). (g) The TFRC levels in AML cells obtained from progressor and non-progressor groups among AML patients administered combined venetoclax and azacitidine treatment (GSE155431, n=6 in the non-progressor group and n=3 in the progressor group). (h) TPM expression of TFRC in AML cells of non-responder, partial responder and responder groups among AML patients treated with fedratinib (GSE199455, n=3, n=4, and n=8, respectively). TPM, transcripts per million; CR, complete remission; non-CR, noncomplete remission.
